# Machine learning-driven identification of shared and disease-specific mitochondria-related genes in COPD, NSCLC, and NSCLC with COPD

**DOI:** 10.1016/j.isci.2026.114857

**Published:** 2026-01-29

**Authors:** Siyu Wu, Zelin Chen, Tongxinwei Sun, Beibei Song, Xinxiu Liu, Liwen Zhang, Jing Li, Haoran Lu, Wenhui Song, Aihong Meng

**Affiliations:** 1 Department of Pulmonary and Critical Care Medicine, The Second Hospital of Hebei Medical University, Shijiazhuang 050000, China; 2 Hebei Key Laboratory of Respiratory Critical Care Medicine, Shijiazhuang 050000, China

**Keywords:** Health sciences, Medicine, Medical specialty, Internal medicine, Oncology, Respiratory medicine

## Abstract

Chronic obstructive pulmonary disease (COPD) and non-small-cell lung cancer (NSCLC) often coexist; here, the shared mitochondrial drivers were investigated. Serum from 30 subjects (seven controls, nine COPD, eight NSCLC, and six NSCLC with COPD) underwent RNA-seq, integrated with 1,136 MitoCarta 3.0-derived mitochondrial-related genes (MRGs). DESeq2 identified 25, 124, and 58 mitochondria-related differentially expressed genes (MR-DEGs) in COPD, NSCLC, and their comorbidity, respectively, with 15 and 58 overlapping genes in relevant pairs. SVM-RFE selected two biomarker sets (3-gene and 5-gene), showing excellent diagnostic performance via ROC (AUC 0.89–0.92) and accurate multivariate logistic regression models. GSEA highlighted immune-inflammatory and oxidative phosphorylation pathways; CIBERSORT revealed altered immune cell proportions (e.g., elevated monocytes in COPD) with biomarker-immune cell correlations. CTD linked *BID/COX7A2* to NSCLC, and DGIdb identified metformin/ME-344 as potential drugs. These mitochondrial gene signatures, validated in blood as robust classifiers of COPD, NSCLC, and their overlap, simultaneously furnish diagnostic biomarkers and actionable therapeutic targets, underscoring the translational value of mitochondria-immune crosstalk.

## Highlights

• Blood-based mitochondrial gene signatures distinguish COPD, NSCLC and NSCLC with COPD
• *NDUFB6/BID/COX7A2* yield ROC-AUC >0.7 across all groups; validated by qPCR
• Machine-learning models reveal mitochondria-immune crosstalk in COPD, NSCLC & NSCLC with COPD
• Metformin & *ME-344* nominated to target *NDUFB6/NDUFA1* via drug-repurposing

## Graphical abstract

**Figure:**
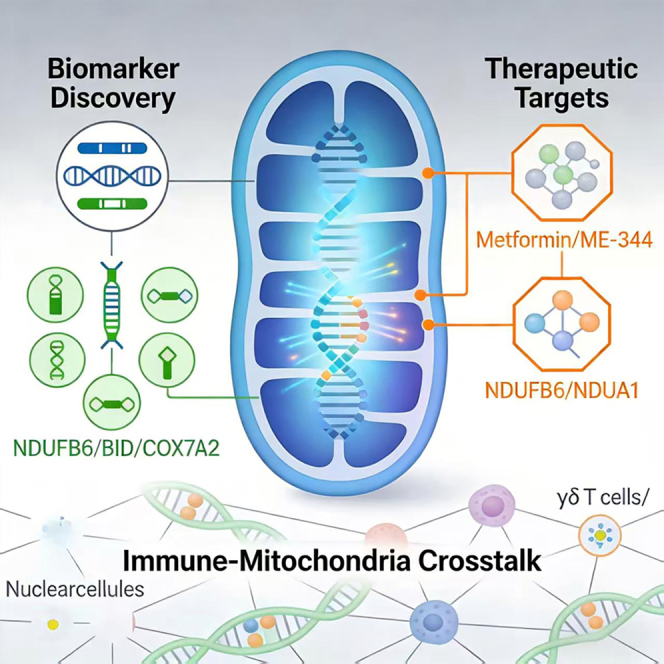

## Introduction

COPD is a common lung disease that has become the third leading cause of death globally. The risk factors for this disease include genetic factors, smoking, and airway inflammation.^1^ Its symptoms include mucus hypersecretion and airway obstruction, and COPD can further develop into conditions including cor pulmonale and respiratory failure. The mortality and disability rates associated with this disease are relatively high.^2^ Compared to healthy individuals, COPD patients also have a higher risk of other diseases, such as lung cancer.^3^^,^^4^^,^^5^ However, COPD patients are primarily treated with medications to alleviate symptoms, but if COPD rapidly worsens, new medications are usually required for treatment.

Lung cancer is the most common cancer in the world, with NSCLC accounting for 80%–85% of all lung cancer cases.^6^^,^^7^ It is a collection of various types of cancers originating from lung epithelium, except for small-cell lung cancer (SCLC). NSCLC includes squamous cell carcinoma, adenocarcinoma, adenosquamous carcinoma, and large cell carcinoma. Compared to SCLC, the cancer cells in NSCLC grow and divide more slowly, and metastasis occurs later. Risk factors for the development of NSCLC include smoking, pulmonary fibrosis, tuberculosis, radiation therapy, and COPD.^8^ Studies have shown that COPD patients have an increased risk of developing lung cancer, and up to 70% of smokers are diagnosed with COPD before being diagnosed with lung cancer.^9^^,^^10^ During the occurrence and development of NSCLC, the overactivation of oncogenes or mutations in tumor suppressor genes can trigger the disease.^11^ Because of COPD, patients have reduced tolerance to lung cancer treatment, making treatment more difficult. For NSCLC, surgery and chemotherapy are currently the main treatment options, but there are often serious adverse reactions to chemotherapy.^12^^,^^13^ New therapeutic strategies for NSCLC are therefore urgently needed.^14^ Research on the pathogenesis and molecular characteristics of NSCLC with COPD remains limited, with many questions unanswered. These unresolved issues restrict a deeper understanding and effective treatment of NSCLC with COPD. Therefore, it is urgent to study the pathogenesis and molecular features of COPD in NSCLC in order to provide stronger support for clinical diagnosis and treatment.

As extranuclear organelles with their own genetic material, mitochondria sustain cellular homeostasis by participating in signal transduction, regulating cell proliferation, apoptosis, metabolism, and immune responses, and governing energy metabolism through ATP generation, reactive oxygen species production via oxidative phosphorylation, and intracellular calcium homeostasis.^15^^,^^16^^,^^17^ Mitochondria promote NSCLC progression through multiple mechanisms. For example, MTCH2 overexpression maintains high mitochondrial function to drive NSCLC cell growth^18^; AIM2 (highly expressed in NSCLC with poor prognosis) colocalizes with mitochondria to boost tumor growth, while its knockdown upregulates MFN2 via enhancing mitochondrial fusion.^19^ A study using GEO dataset GSE57148 identified 12 MitoDEGs, building a diagnostic model with ERN1, FASTK, HIGD1B, NDUFA7, and NDUFB7. In COPD, ERN1 is upregulated and the four others are downregulated; HIGD1B/NDUFB7 mRNA is reduced in COPD mice, clarifying mitochondria-immunity interaction.^20^

Recent bioinformatics studies have explored the molecular overlaps between COPD and NSCLC subtypes,^21^ highlighting the complex interplay of disease mechanisms through integrative approaches.^22^^,^^23^ The correlation between COPD, NSCLC, and NSCLC with COPD in terms of disease onset and development was investigated in our study. We explored the biological pathways, immune characteristics, and drug predictions involved in MRG-related biomarkers via transcriptome sequencing, thus providing new insights into the treatment of COPD, NSCLC, and NSCLC with COPD, which would improve the prognosis of patients with COPD, NSCLC, and NSCLC with COPD.

## Results

### Differential expression analysis

**Figure 1:**
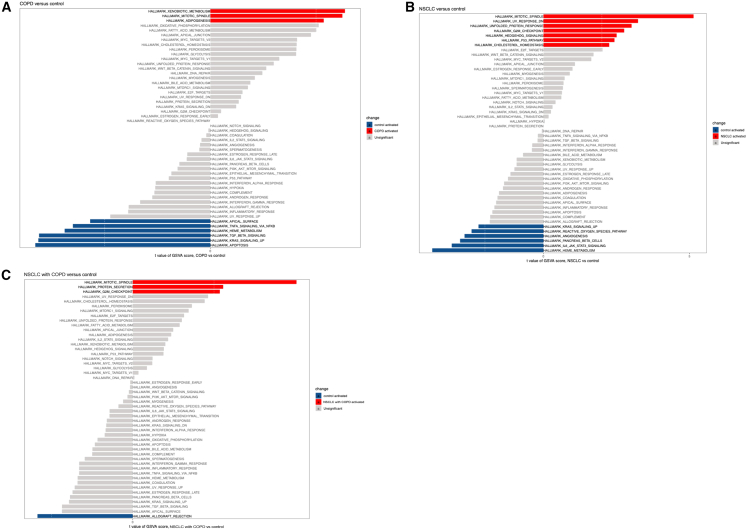
Differential expression and functional enrichment analysis (A–C) Gene set variation analysis (GSVA) enrichment scores comparing (A) COPD versus controls, (B) NSCLC versus controls, and (C) NSCLC with COPD versus controls, highlighting distinct biological pathway alterations across disease states.

**Figure 2:**
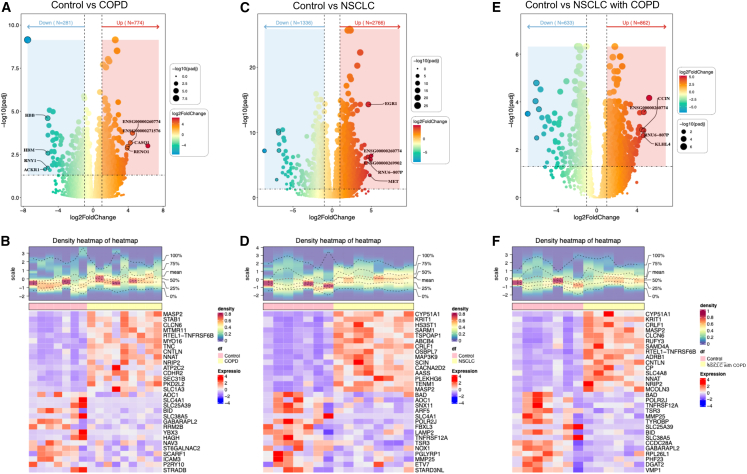
Identification of DEGs Volcano plot of the distribution of DEGs1 between COPD and control group (A), DEGs2 between NSCLC and control group (C), DEGs3 between NSCLC with COPD and control group (E), red indicates upregulated genes; blue indicates downregulated genes. Heatmap of DEGs1 between COPD and control group (B), DEGs2 between NSCLC and control group (D), and DEGs3 between NSCLC with COPD and control group (F), this figure plots the top 15 upregulated genes and top 15 downregulated genes.

To explore MRGs and their potential roles as biomarkers in COPD, NSCLC, and NSCLC with COPD, we collected seven control samples, nine COPD samples, eight NSCLC samples, and six NSCLC with COPD samples for RNA sequencing and a series of data analyses (Table 1) with experimental validation, the specific workflow was illustrated in Figure S1. First, to investigate global differences in molecular functions between disease groups and controls, we performed GSVA analyses and revealed the following distinct pathway alterations. Pathways including xenobiotic metabolism, mitotic spindle, and adipogenesis were activated, while pathways including apical surface, TNFA signaling via NF-κB, heme metabolism, TGF beta signaling, KRAS signaling, and apoptosis were suppressed in the COPD group compared with control group (Figure 1A). The NSCLC group showed enrichment in mitotic spindle, UV response, unfolded protein response, G2/M checkpoint, Hedgehog signaling, p53 pathway, and cholesterol homeostasis, with suppression of KRAS signaling, reactive oxygen species (ROS) pathway, angiogenesis, pancreatic β-cell activation, IL6/JAK/STAT3 signaling, and heme metabolism in the comparison with control group (Figure 1B). NSCLC with the COPD group displayed activated pathway activities in mitotic spindle, protein secretion, and G2/M checkpoint, alongside suppressed pathway activity in allograft rejection relative to control group (Figure 1C). For further screening of DEGs between groups, using the criteria of adjusted *p* value < 0.05 and |log_2_ FC| > 1, we identified 1,055 DEGs in COPD (774 upregulated, 281 downregulated; Figures 2A and 2B), 4,102 in NSCLC (2,706 upregulated, 1,336 downregulated; Figures 2C and 2D), and 1,495 in NSCLC with COPD (862 upregulated, 633 downregulated; Figures 2E and 2F). We identified DEGs in COPD vs. control, NSCLC vs. control, and NSCLC with COPD vs. control as DEG1, DEG2, and DEG3, correspondingly. Heatmaps emphasize the 15 most significantly upregulated and downregulated genes in each group.

### Enrichment analysis of DEG functions

**Figure 3:**
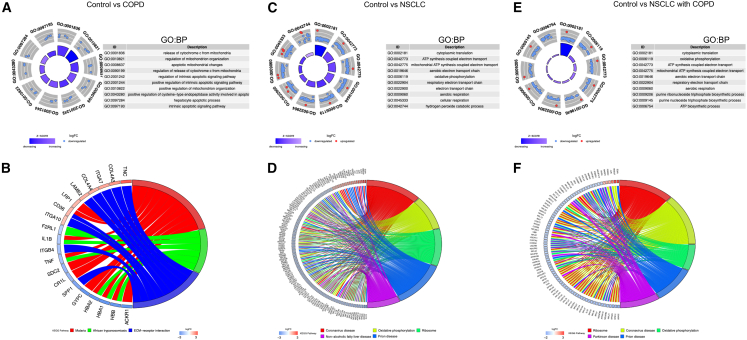
GO and KEGG analysis of DEGs The top ten biological process terms of GO analysis of the DEGs1 between COPD and control group (A), DEGs2 between NSCLC and control group (C), DEGs3 between NSCLC with COPD and control group (E). Chord diagram of KEGG analysis of DEGs1 between COPD and control group (B), DEGs2 between NSCLC and control group (D), and DEGs3 between NSCLC with COPD and control group (F).

To examine the biological functions and pathways linked to DEGs, enrichment analyses for GO and KEGG were performed. For DEG1 (COPD vs. controls), GO terms highlighted mitochondria-related functions such as mitochondrial changes during apoptosis and mitochondrial genome release (Figures 3A, S2A, and S2B), suggesting that mitochondrial dysfunction may contribute to COPD. KEGG pathways were enriched in extracellular matrix receptor interactions and pathogenic infections (Figure 3B). For DEG2 (NSCLC vs. controls), GO terms included energy metabolism-related functions (e.g., ATP synthesis, respiratory electron transport, and oxidative phosphorylation), linking NSCLC to mitochondrial energy metabolism (Figures 3C, S2C, and S2D). KEGG pathways involved oxidative phosphorylation, ribosome function, and viral infections (e.g., coronavirus) (Figure 3D). For DEG3 (NSCLC with COPD vs. controls), GO terms focused on energy metabolism (e.g., ATP synthesis, electron transport chain, and anaerobic respiration), implicating mitochondrial function in NSCLC with COPD (Figures 3E, S2E, and S2F). KEGG pathways overlapped with those of DEG2, including oxidative phosphorylation and viral infections (Figure 3F). From the above results, we found that those DEGs are closely related to cellular energy and mitochondria. Therefore, we speculate that these DEGs may participate in the occurrence of the COPD, NSCLC, and NSCLC with COPD by affecting mitochondrial function.

### Screening of MR-DEGs

**Figure 4:**
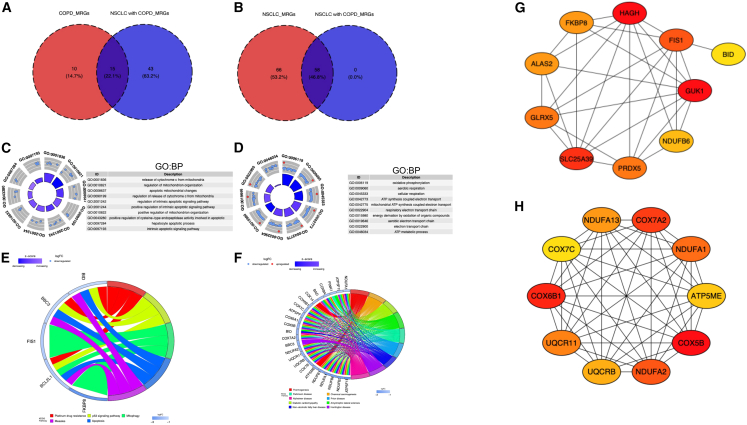
Identification and Functional Networks of Common Genes Sets (A and B) Venn diagrams illustrating overlap in common genes set 1 (A) and common genes set 2 (B). (C and D) Biological process (BP) terms from GO analysis of common genes set 1 (C) and common genes set 2 (D). (E and F) KEGG pathway enrichment of common genes set 1 (E) and common genes set 2 (F). (G and H) PPI networks of top 10 hub genes identified from common genes set 1 (G) and common genes set 2 (H).

To identify MR-DEGs across the three comparison groups, we intersected DEG1, DEG2, and DEG3 with 1,136 MRGs, which was obtained from MitoCarta 3.0, yielding 25 MR-DEGs in COPD, 124 in NSCLC, and 58 in NSCLC with COPD (Figures S3A–S3C). To identify shared genes among these diseases, we first intersected COPD and NSCLC with COPD-associated MR-DEGs, obtaining a common gene set 1 comprising 15 genes. Next, we intersected NSCLC and NSCLC with COPD-associated MR-DEGs, resulting in common gene set 2 containing 58 genes (Figures 4A and 4B). Functional enrichment analysis (GO and KEGG) of common gene set 1 revealed that the top ten GO terms were significantly associated with mitochondrial processes, including *mitochondrial changes during apoptosis* and *release of mitochondrial genome* (Figures 4C, S3D, and S3E), suggesting that mitochondrial dysfunction may be a key factor in COPD and NSCLC with COPD pathogenesis. Furthermore, the KEGG analysis showed that the genes involved mainly in apoptosis, mitophagy, and the p53 signaling pathway (Figure 4E). For common gene set 2, GO analysis highlighted pathways closely linked to mitochondrial function, such as oxidative phosphorylation, cellular respiration, ATP synthesis, and electron transport chain activity (Figures 4D, S3F, and 3G). KEGG enrichment revealed associations with metabolic disorders, including diabetic complications, fatty liver disease, and non-alcoholic fatty liver disease (Figure 4F). To elucidate the regulatory roles of those genes, we constructed protein-protein interaction (PPI) networks for both common gene set 1 and 2 (Figures S3H and S3I) and identified the top ten hub genes as candidate key regulators. For common gene set 1, the top ten hub genes were FKBP8, HAGH, FIS1, *BID*, GUK1, *NDUFB6*, *PRDX5*, SLC25A39, GLRX5, and ALAS2. For common gene set 2, the top ten hub genes included NDUFA13, *COX7A2*, *NDUFA1*, ATP5ME, COX5B, NDUFA2, *UQCRB*, *UQCR11*, COX6B1, and *COX7C* (Figures 4G and 4H). These findings suggest that mitochondrial dysfunction, mediated by distinct gene regulatory networks, may contribute to disease progression in COPD, NSCLC, and NSCLC with COPD through mechanisms involving apoptosis, metabolic dysregulation, and oxidative stress.

### Machine learning-based biomarker identification and diagnostic performance evaluation

**Figure 5:**
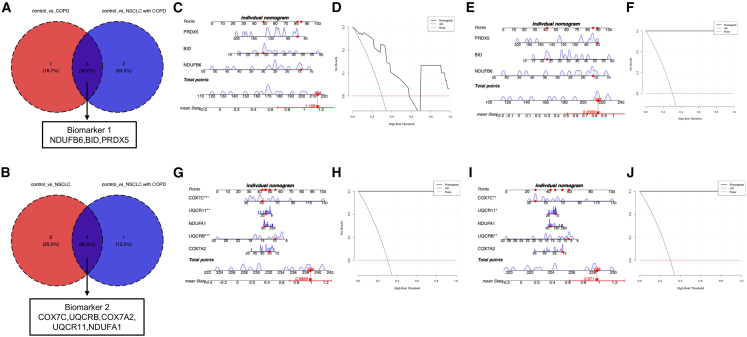
Diagnostic model construction and validation (A and B) Venn diagrams of biomarker 1 (A) and biomarker 2 (B) selection. (C and E) Nomograms integrating biomarker1 expression to predict disease probability for COPD (C) and NSCLC with COPD (E). (G and I) Nomograms integrating biomarker2 expression to predict disease probability for NSCLC (G) and NSCLC with COPD (I). (D and F) Decision curve analysis (DCA) evaluating clinical utility of biomarker 1-based models for COPD (D) and NSCLC with COPD (F). (H and J) Decision curve analysis (DCA) evaluating clinical utility of biomarker 2-based models for for NSCLC (H) and NSCLC with COPD (J).

To identify robust diagnostic biomarkers, we employed the SVM-RFE algorithm to analyze the above-mentioned two candidate common gene sets in our transcriptomic dataset. For the common gene set 1, optimal classification of COPD versus controls (RMSE = 0.3360) identified four key biomarkers: *NDUFB6*, *BID*, FKBP8, and *PRDX5* (Figure S4A), while NSCLC with COPD versus controls (RMSE = 0.3220) yielded five discriminative genes: *NDUFB6*, *BID*, GLRX5, *PRDX5*, and SLC25A39 (Figure S4B). The intersection of these results produced biomarker set 1 (*NDUFB6*, *BID*, and *PRDX5*), demonstrating dual diagnostic capability for both COPD and NSCLC with COPD (Figure 5A). Similarly, analysis of the common gene set 2 revealed seven NSCLC-specific biomarkers (*COX7A2*, *UQCRB*, COX6B1, *NDUFA1*, COX5B, *UQCR11*, and *COX7C*; RMSE = 0.4521) (Figure S4C) and six NSCLC with COPD-specific markers (*COX7C*, *UQCRB*, *COX7A2*, *UQCR11*, NDUFA13, and *NDUFA1*; RMSE = 0.4364) (Figure S4D), with their overlap forming biomarker set 2 (*COX7C*, *UQCRB*, *COX7A2*, *UQCR11*, and *NDUFA1*) for both NSCLC and NSCLC with COPD detection (Figure 5B). ROC analysis confirmed excellent diagnostic performance for both biomarker sets (AUC > 0.7 for all comparisons) (Figures S4E–S4H). Subsequent multivariate logistic regression models incorporating these biomarkers showed strong predictive accuracy, with calibration errors of 0.107 (COPD model), 0.063 (NSCLC with COPD model) for biomarker set 1(Figures S4I and S4J), and 0.013 (NSCLC model), 0.063 (NSCLC with COPD model) for biomarker set 2 (Figures S4K and S4L). Nomogram and DCA further validated the potential clinical utility of diagnostic models of biomarker set 1 to distinguish COPD or NSCLC with COPD from control (Figures 5C–5F), as well as the biomarker set 2 to distinguish NSCLC or NSCLC with COPD from control (Figures 5G–5J). These findings establish MRGs signatures as powerful diagnostic tools for respiratory diseases, with particular promise for distinguishing between disease subtypes and controls.

### Functional characterization of biomarkers

**Figure 6:**
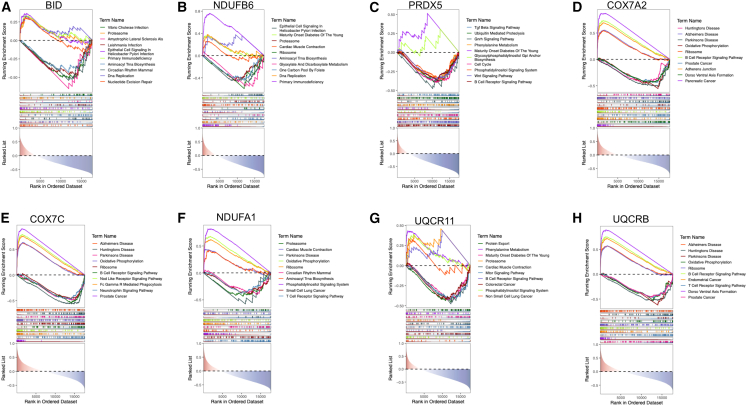
Single-gene GSEA of biomarkers (A–C) Enriched pathways associated with *BID* (A), *NDUFB6* (B), and *PRDX5* (C) in biomarker 1. (D–H) Enriched pathways associated with *COX7A2* (D), *COX7C* (E), *NDUFA1* (F), *PRDX5* (G), and *UQCRB* (H) in biomarker 2.

To explore potential biological pathways linked to our identified biomarkers, gene set enrichment analysis (GSEA) was utilized. Unlike traditional GO enrichment analysis, GSEA evaluates how predefined gene sets are distributed in phenotype-correlated gene rankings, thereby capturing subtle but coordinated biological changes that might be missed by differential expression analysis alone. For biomarker set 1 (*NDUFB6*, *BID*, and *PRDX5*), single-gene GSEA revealed significant enrichment in multiple immune-inflammatory cascades, such as primary immunodeficiency and B cell receptor-associated signaling pathways (Figures 6A–6C). These biomarkers were also associated with fundamental cellular processes such as DNA repair and proteasome function. These findings suggest that dysregulation of these three key genes may contribute to COPD and NSCLC with COPD pathogenesis by disrupting immune homeostasis and compromising basic cellular physiology. Similarly, analysis of biomarker set 2 (*COX7C*, *UQCRB*, *COX7A2*, *UQCR11*, and *NDUFA1*) demonstrated comparable enrichment patterns in immune-inflammatory pathways, along with involvement in essential cellular maintenance processes (Figures 6D–6H). The consistent association of both biomarker sets with immune dysregulation and cellular stress responses supports their potential role in mediating disease-specific pathological changes through these shared mechanisms.

### Immune infiltration landscape analysis

**Figure 7:**
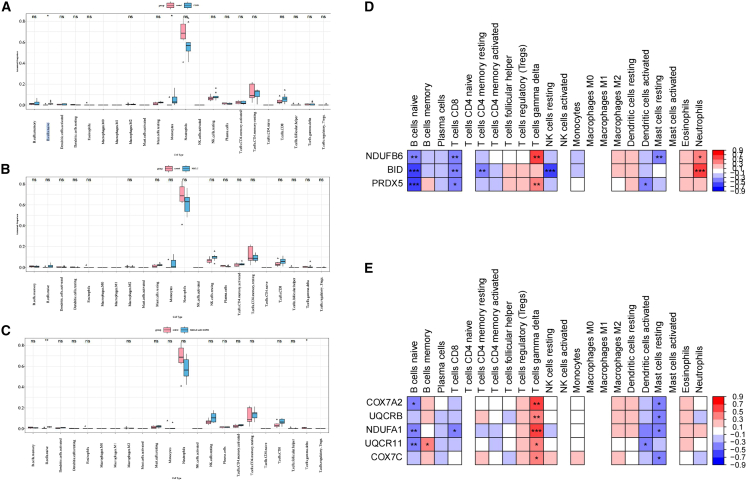
Immune microenvironment associations between biomarkers and immune cells (A–C) Differential infiltration of immune cells between COPD vs. control (A), NSCLC vs. control (B), and NSCLC with COPD vs. control (C). Data are represented as median with interquartile range. Statistical significance was determined by the Wilcoxon rank-sum test. (D and E) Correlations between immune cells and biomarker 1 (D), and between immune cells and biomarker 2 (E) (|r| > 0.3, *p* < 0.05). Correlation coefficients were calculated using Spearman’s correlation analysis. ∗*p* < 0.05, ∗∗*p* < 0.01, ∗∗∗*p* < 0.001. ns, not significant.

To characterize immune microenvironment differences between disease groups and controls, we quantified infiltration levels of 22 immune cell types and visualized their relative abundance using stacked bar plots (Figure S5A). Comparative analysis revealed significant alterations in COPD versus controls, with elevated monocytes and naive B cells (Wilcoxon test, *p* < 0.05; Figure 7A), while NSCLC showed no significant immune infiltration differences (Figure 7B). NSCLC with COPD exhibited distinct changes including increased naive B cells and decreased regulatory T cells compared to controls (*p* < 0.05; Figure 7C). Subsequent Spearman correlation analysis of intercellular relationships (Figure S5B) and biomarker-immune cell interactions demonstrated significant associations (|r| > 0.3, *p* < 0.05): For biomarker set 1, *NDUFB6* correlated with naive B cells, CD8^+^ T cells, γδ T cells, resting mast cells, and neutrophils; *BID* associated with them as well as resting memory CD4^+^ T cells and resting NK cells, and *PRDX5* linked to naive B cells, CD8^+^ T cells, γδ T cells, and activated dendritic immune cells(Figure 7D). Biomarker set 2 showed *COX7A2* correlations with naive B cells, γδ T cells, and resting mast cells; *UQCRB* associations with γδ T cells and resting mast cells; *NDUFA1* connections to naive B cells, γδ T cells, resting mast cells, and CD8^+^ T cells; *UQCR11* interactions with naive B cells, memory B cells, γδ T cells, resting mast cells, and stimulated dendritic cells; and *COX7C* correlations with γδ T cells and resting mast cells (Figure 7E). In summary, we believe that on the one hand, the biomarkers regulate mitochondrial function, and on the other hand, they might affect the immune microenvironment.

### Transcriptional regulation and drug network of biomarkers

**Figure 8:**
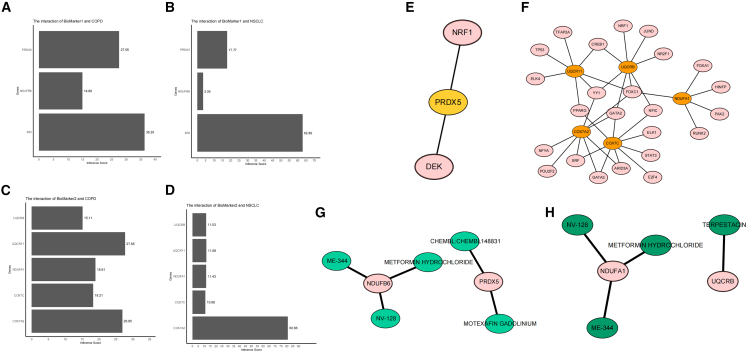
Biomarker-disease interaction networks (A and B) Correlations between COPD (A), NSCLC (B), and biomarker 1. (C and D) Correlations between COPD (C), NSCLC (D), and biomarker 2. (E and F) Regulatory networks of transcription factors targeting biomarker set 1 (E) and biomarker set 2 (F). (G–H) Interaction networks between drugs and biomarker 1 (G), and between drugs and biomarker 2 (H).

To elucidate biomarker-disease relationships, we analyzed associations between biomarker sets 1/2 and COPD/NSCLC using the Comparative Toxicogenomics Database (CTD) inference scores, revealing particularly strong correlations of *BID* and *COX7A2* with NSCLC (Figures 8A–8D). Furthermore, transcription factor (TF) prediction via NetworkAnalyst and JASPAR database identified NRF1 and DEK as potential regulators of *PRDX5* in biomarker set 1 (Figure 8E), while 24 TFs were predicted to regulate the five genes comprising biomarker set 2 (Figure 8F). Drug-gene interaction screening through DGIdb database yielded three therapeutic candidates targeting *NDUFB6* (ME-344, NV-128, METFORMIN HYDROCHLORIDE) and two therapeutic candidates targeting *PRDX5* (CHEMBL148831, MOTEXAFIN GADOLINIUM) in biomarker set 1 (Figure 8G), along with four agents targeting *NDUFA1* (ME-344, NV-128, METFORMIN HYDROCHLORIDE) and one agents targeting *UQCRB* (TERPESTACIN) in biomarker set 2 (Figure 8H), thereby identifying repurposable drugs with potential efficacy against these respiratory disorders.

### Expression and validation of biomarkers

**Figure 9:**
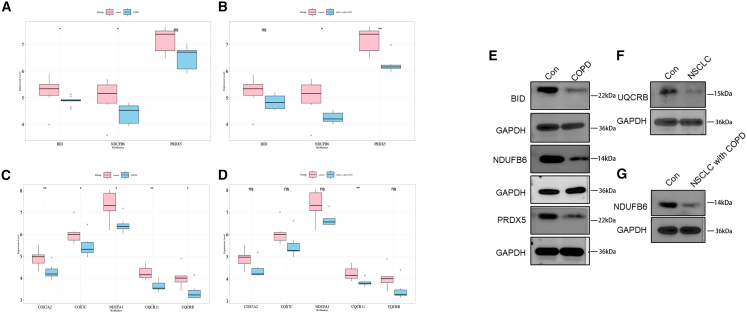
Biomarker expression validation (A and B) Expression differences of biomarker 1 between control vs. COPD (A, *n* = 7 for control, *n* = 9 for COPD) and control vs. NSCLC with COPD (B, *n* = 7 for control, *n* = 6 for NSCLC with COPD). (C and D) Expression differences of biomarker 2 between control vs. NSCLC (C, *n* = 7 for control, *n* = 8 for NSCLC) and control vs. NSCLC with COPD (D, *n* = 7 for control, *n* = 6 for NSCLC with COPD). Data are represented as median with interquartile range. Statistical significance was determined by Wilcoxon rank-sum test. ∗*p* < 0.05, ∗∗*p* < 0.01, ∗∗∗*p* < 0.001; ns, not significant. (E–G) Western blot analysis of biomarker expression levels between control vs. COPD (E), control vs. NSCLC (F), and control vs. NSCLC with COPD (G). GAPDH was used as the loading control.

To validate biomarker expression patterns, Wilcoxon rank-sum tests revealed that in biomarker set 1, both *BID* and *NDUFB6* were significantly downregulated in COPD and NSCLC with COPD groups compared to controls, while *PRDX5* showed no significant difference in COPD (though approaching significance, *p* ≈ 0.05) but was significantly reduced in NSCLC with COPD (Figures 9A and 9B); for biomarker set 2, all five biomarkers exhibited significant differential expression in NSCLC versus controls (Figure 9C), whereas in NSCLC with COPD only *UQCR11* reached significance with the other four biomarkers approaching the threshold (*p* ≈ 0.05) (Figure 9D). We have performed Western blot validation for key biomarkers as shown in Figures 9E–9G. The Western blot results confirm the downregulation of *BID*, *NDUFB6*, and *PRDX5* in COPD compared with control (Figure 9E),the downregulation of *UQCRB* in NSCLC compared with control (Figure 9F), and the downregulation of *NDUFB6* in NSCLC with COPD compared with control (Figure 9G), these western blot results consistent with our RNA sequencing findings as shown in Figures 9A–9D.

## Discussion

In the study of biomarkers for COPD, NSCLC, and NSCLC with COPD, a systematic workflow was used for screening and validation: DEGs were identified in each disease group, intersected with 1,136 MRGs from MitoCarta 3.0 to get MR-DEGs, and two shared gene sets were obtained (15 genes between COPD and NSCLC with COPD; 58 genes between NSCLC and NSCLC with COPD). Two diagnostic biomarker sets were screened: set 1 (*NDUFB6*, *BID*, and *PRDX5*) for distinguishing COPD from NSCLC with COPD, and set 2 (five genes including *COX7C*, *UQCRB*) for distinguishing NSCLC from NSCLC with COPD. ROC analysis showed their AUC exceeded 0.7; multivariate logistic regression models had low calibration errors, and nomogram and DCA confirmed clinical application potential.

Previous studies confirmed that MRGs are enriched in mitochondrial apoptosis pathways, with BCL2 family and caspase cascade regulating airway injury and tumor progression.^24^ Further research found MRG-mediated abnormal mitophagy and imbalanced ROS metabolism may drive COPD progression to NSCLC,^25^ and these pathways are active in heart failure^26^ and neurodegenerative diseases,^27^ indicating mitochondrial dysfunction’s cross-disease pathological basis. MRGs promote chronic inflammation to immunosuppression by modulating T cell infiltration and myeloid immune cell polarization (e.g., myeloid-derived suppressor cells [MDSCs], regulatory T cells [Tregs])^28^^,^^29^; mitochondrial ROS links inflammation and tumor immune escape via NF-κB/STING pathway.^30^^,^^31^ Additionally, non-coding RNAs (e.g., miR-34a, MALAT1) and epigenetic modifications (e.g., DNA methylation) regulate MRG expression and mitochondrial homeostasis.^32^^,^^33^ Immune microenvironment analysis showed associations between biomarkers and immune cells. Quantification of 22 immune cells revealed COPD had higher monocytes and naive B cells than controls (*p* < 0.05); NSCLC with COPD had increased naive B cells and decreased Tregs (*p* < 0.05); NSCLC had no significant differences from controls. Spearman correlation analysis showed: in COPD and NSCLC with COPD, biomarker set 1 correlated with naive B cells and CD8^+^ T cells. Naive B cell function relies on mitochondrial energy, and biomarker-induced mitochondrial dysfunction impairs their activation, weakening humoral immunity,^34^ consistent with COPD’s airway mucosal immunity impairment and NSCLC with COPD’s defective antibody-mediated clearance. CD8^+^ T cell killing depends on ROS,^35^ and abnormal biomarkers (e.g., altered *PRDX5*) disrupt ROS metabolism, impairing activation or inducing exhaustion. In NSCLC and NSCLC with COPD, biomarker set 2 associated with γδ T cells, whose tumor killing relies on mitochondrial metabolites^36^; abnormal Set 2 genes disrupt mitochondrial function, impairing γδ T cell function and facilitating tumor escape.^37^

### Mechanistic insights: How key biomarkers link mitochondrial dysfunction to immune dysregulation

Our identified biomarkers orchestrate a complex interplay between mitochondrial dysfunction and immune dysregulation through multiple converging mechanisms. The respiratory chain components *NDUFB6*, *NDUFA1* (complex I), *UQCRB*, *UQCR11* (complex III), and *COX7A2*, *COX7C* (Complex IV) directly modulate oxidative phosphorylation (OXPHOS) efficiency. Recent studies demonstrate that selective complex I subunit deficiency enhances tumor immunogenicity by inducing pyruvate dehydrogenase-dependent acetyl-CoA accumulation, which promotes histone H3K27 acetylation and upregulates MHC class I antigen presentation machinery.^38^ Similarly, impaired complex III function diminishes mitochondrial respiration and ATP production, driving CD8^+^ T cells toward an exhausted phenotype characterized by reduced proliferation and memory formation.^35^ This metabolic collapse is further exacerbated by mitochondrial DNA mutations transferring from cancer cells to tumor-infiltrating lymphocytes, perpetuating immune dysfunction through defective mitophagy.^39^ *BID* bridges mitochondrial apoptosis and immune cell survival. Upon caspase-8 activation, truncated *BID* (tBID) translocates to mitochondria, inducing outer membrane permeabilization and cytochrome *c* release, thereby modulating CD8^+^ T cell apoptotic sensitivity.^40^ *PRDX5*, localized across mitochondria, peroxisomes, and cytosol, serves as a critical antioxidant defense mechanism. By reducing hydrogen peroxide, alkyl hydroperoxides, and peroxynitrite, *PRDX5* protects against ROS-induced mitochondrial damage and maintains redox homeostasis essential for immune cell function.^41^ Collectively, downregulation of these biomarkers in COPD, NSCLC, and NSCLC with COPD patients creates a “metabolic-immune crisis.”: impaired OXPHOS reduces ATP availability for T cell activation and proliferation, accumulated ROS triggers oxidative stress-mediated immune cell exhaustion, and dysregulated apoptotic pathways compromise immunosurveillance.^42^ This mechanistic framework positions mitochondrial restoration as a promising therapeutic axis to reinvigorate anti-tumor immunity.

### “Mitochondria-immune microenvironment” interaction

From a shared perspective on “mitochondrial-immune microenvironmental interactions”, the primary function of γδ T cells are well-recognized and these cells act as a core associated component across all three diseases: in COPD, biomarker-regulated mitochondrial dysfunction causes metabolic disorders and oxidative stress, altering γδ T cell activation and cytokine secretion (e.g., excessive IL-17 release). This exacerbates chronic airway inflammation and tissue damage.^43^ In NSCLC, mitochondrial dysfunction (mediated by biomarkers) impairs γδ T cell metabolism and signaling, suppressing anti-tumor activity and enabling tumor immune escape.^44^ In NSCLC with COPD, the coexistence of COPD-related airway inflammatory microenvironment and NSCLC-related tumor microenvironment leads to more complex interactions between biomarker-regulated mitochondria and γδ T cells (likely overlapping or synergistic mechanisms from both diseases), further driving disease progression.

### Potential regulatory mechanisms and drug repurposing transcriptional regulation

Database analysis showed: *PRDX5* (in biomarker set 1) is regulated by two TFs (NRF1, DEK); biomarker set 2 is regulated by 24 TFs, with *COX7A2* showing strong correlation with NSCLC. Through targeted drug prediction, via the DGIdb database, repurposable drugs were identified: three drugs targeting *NDUFB6* (e.g., metformin) in biomarker set 1; two drugs targeting *PRDX5* (biomarker set 1); four drugs targeting *NDUFA1* (biomarker set 2); one drug targeting *UQCRB* (biomarker set 2). Wilcoxon rank-sum test validated the differential expression of these biomarkers between disease and control groups, further supporting their reliability. Therapeutic implications drugs like metformin improve mitochondrial function via the AMPK/mTOR pathway; gene-specific killing effects in the context of LKB1 mutations provide a basis for personalized treatment^45^; potential applications of novel mitochondria-targeted drugs (e.g., MitoQ) in NSCLC with COPD models expand the clinical translation prospects of MRGs.

### Conclusion

This research represents the first attempt to systematically clarify the pivotal role of mitochondrial dysfunction in COPD, NSCLC, and NSCLC with COPD, revealing differences and commonalities of MRGs across these diseases. Key findings include the followings: biomarkers disrupt immune homeostasis by interfering with inflammatory pathways and regulating mitochondrial function to impact immune cell activity, and γδ T cells are a critical node in “mitochondria-immune microenvironment” interactions across all three diseases. The identified biomarkers provide a theoretical basis for early disease diagnosis and novel targets for mechanistic research.^46^^,^^47^ The results not only enhance understanding of COPD and NSCLC comorbidity mechanisms but also offer insights for diagnosing and treating other mitochondria-related systemic diseases,^48^ advancing precision medicine for overlapping respiratory diseases.

### Translational implications and future directions

The identified mitochondria-related biomarker sets hold substantial translational potential across multiple clinical domains. Diagnostically, the validated signatures (biomarker set 1: *NDUFB6*, *BID*, *PRDX5*; biomarker set 2: *COX7C*, *UQCRB*, *COX7A2*, *UQCR11*, *NDUFA1*) demonstrate robust performance (AUC >0.7), offering non-invasive serum-based alternatives for early disease detection and differential diagnosis. The nomogram-based risk scoring systems provide clinically actionable tools for patient stratification and personalized screening protocols in high-risk populations. For therapeutic targeting, drug-gene interaction networks reveal repurposing opportunities for existing agents—particularly metformin and ME-344 targeting complex I subunits (*NDUFB6*/*NDUFA1*)—warranting urgent preclinical validation. The mechanistic link between biomarker dysregulation and immune microenvironment alterations (γδ T cell dysfunction, naive B cell activation, and Treg depletion) suggests combination strategies targeting both mitochondrial restoration and immune checkpoint modulation may synergistically enhance efficacy. Novel mitochondria-targeted compounds (e.g., MitoQ) could be integrated into standard regimens to mitigate treatment-associated toxicity. Regarding clinical decision-making support, biomarker-immune cell correlation patterns could inform treatment selection—for instance, patients with high *PRDX5* expression and elevated naive B cells might preferentially respond to antioxidant-immunomodulatory combinations. Longitudinal biomarker monitoring could enable real-time therapeutic response assessment and early progression detection. Future priorities include the followings: (1) multicenter validation across diverse populations; (2) mechanistic dissection via CRISPR-based experiments and animal models to establish causality; (3) prospective studies tracking biomarker dynamics through disease progression; (4) proteomic validation correlating mRNA-protein expression; (5) drug efficacy testing in patient-derived organoids and clinical trials; and (6) development of companion diagnostics for routine clinical implementation. These translational efforts could bridge mechanistic insights to clinical practice, ultimately improving patient outcomes.

### Limitations of the study

Despite valuable findings on mitochondria-related biomarkers in COPD, NSCLC, and NSCLC with COPD, the study has limitations that require further refinement: small, single-center sample size may introduce bias in DEG screening and biomarker diagnostic efficacy assessment, and regional/population confounding factors reduce result generalizability. Future studies should use larger, multi-center cohorts. Limited biomarker validation is another such limitation, while Wilcoxon rank-sum test confirmed gene expression differences, validation is lacking at the multiple levels (e.g.,.immunohistochemistry) and in prospective clinical studies (to evaluate predictive value for disease progression/treatment response). There mechanistic research is insufficient: causal relationships between biomarkers, mitochondrial function, and immune microenvironment have not been verified via cell experiments (e.g., gene knockout/overexpression) or animal models. Lack of drug validation also needs to be considered: database-predicted drugs (e.g., metformin, TERPESTACIN) have not been tested *in vitro* (cell activity assays) or *in vivo* (animal models) to confirm their regulatory effects on biomarkers or therapeutic efficacy.

## Resource availability

### Lead contact

Further information and requests for resources and reagents should be directed to and will be fulfilled by the lead contact, Aihong Meng (mah123@hebmu.edu.cn).

### Materials availability

This study did not generate new unique reagents.

### Data and code availability

• Raw RNA-seq data and the datasets used in this study are deposited in ScienceDB with the assigned DOI: https://doi.org/10.57760/sciencedb.27559.
• The analysis code is now deposited in ScienceDB (DOI: https://doi.org/10.57760/sciencedb.27559).
• Further information and resource/reagent requests should be directed to A.M. (mah123@hebmu.edu.cn) or S.W. (28403081@hbmu.edu.cn).

## Acknowledgments

We sincerely thank Dr. Rong Chen from the Experimental Center of the North Campus of the Second Hospital of 10.13039/501100012505Hebei Medical University for his full support in all aspects. We are especially grateful to our junior lab mates for their assistance in establishing the database for patients with lung cancer combined with COPD. We also express our deep appreciation to the professors from the Hebei Provincial Institute of Respiratory Diseases for their fruitful academic discussions and guidance. This study was fully or partially supported by the 10.13039/501100014764China International Medical Foundation (grant no. Z-2017-24-2301) and 2020 10.13039/100017959Hebei Provincial Health Commission Medical Science Research Project (no. 20200950), with the recipient being S.W. and the 2026 Government-funded Clinical Medicine Talent Training Project (grant no. ZF2026107), with the recipient being A.M.

## Author contributions

All authors have read and approved the final manuscript and take full responsibility for its content. Using the CRediT (Contributor Roles Taxonomy) framework, individual contributions are specified below: conceptualization, S.W., Z.C., A.M., T.S., B.S., X.L., H.L., and W.S.; design, S.W., Z.C., and A.M.; investigation/acquisition, S.W., Z.C., T.S., B.S., X.L., H.L., J.L., and W.S.; analysis, S.W., Z.C., and A.M.; interpretation of data, S.W. and Z.C.; resources, S.W., Z.C., and A.M.; drafting/revision, S.W. and Z.C.; visualization, S.W., Z.C., and A.M.

## Declaration of interests

The authors declare no competing interests.

## STAR★Methods

### Key resources table

**Table:** 

REAGENT or RESOURCE	SOURCE	IDENTIFIER
**Antibodies**		

Rabbit anti-NDUFB6	CUSABIO	Cat# CSB-PA015653LA01HU; RRID:AB_3719878
Rabbit anti-UQCRB	Abiowell	Cat# AWA61861; RRID:AB_3719879
Rabbit anti-PRDX5	Abiowell	Cat# AWA62829; RRID:AB_3719880
Rabbit anti-BID	Abiowell	Cat# AWA46519; RRID:AB_3719881
Rabbit anti-GAPDH	Abways	Cat# AB0037; RRID:AB_2891315
Goat anti-rabbit HRP	Reports	Cat# S1002; RRID:AB_3662691

**Biological samples**		

Homo sapiens Serum	N/A	N/A

**Chemicals, peptides, and recombinant proteins**		

RNA Reagent kit	Magen Biotech	Cat# AJ15RK01
VAHTS® Universal V8 RNA-seq Library Prep Kit for Illumina	Vazyme	Cat# 7E0761H4
RNA extraction solution	Servicebio	Cat# G3013
Red Blood Cell Lysis Buffer	Servicebio	Cat# G2015
Chloroform Substitute	Servicebio	Cat# G3014
Isopropyl Alcohol	sinopharm chemical reagent Co.,Ltd	Cat# 80109218
Absolute Ethyl Alcohol	sinopharm chemical reagent Co.,Ltd	Cat# 10009218
RNA dissolution solution	Servicebio	Cat# G3029
Water Nuclease-Free	Servicebio	Cat# G4700
SweScript All-in-One RT SuperMix for qPCR (One-Step gDNA Remover)	Servicebio	Cat# G3337
2×Universal Blue SYBR Green qPCR Master Mix	Servicebio	Cat# G3326

**Deposited data**		

Raw RNA-seq data	ScienceDB	https://doi.org/10.57760/sciencedb.27559
The Analysis Code	ScienceDB	https://doi.org/10.57760/sciencedb.27559

**Oligonucleotides**		

GAPDH-F:GGAAGCTTGTCATCAATGGAAATC GAPDH-R:TGATGACCCTTTTGGCTCCC	Servicebio	NM_002046.7
COX7A2-F:GATTGGGCAGAGGACGATAAGC COX7A2-R:AAGCGATTGCTGGGATGACTG	Servicebio	NM_001366292.3
NDUFA1-F:ATCCACAGGTTCACTAACGGGG NDUFA1-R:TAACGATCAACTCCAGAGATGCG	Servicebio	NM_004541.4
UQCRB-F:CAGTGGACCAAATATGAAGAGGAA UQCRB-R:ACTTCTTTGCCCATTCTTCTCTTTC	Servicebio	NM_001199975.3
COX7C-F:GTAGGAGCCACTATGAGGAGGG COX7C-R:AAGGGTGTAGCAAATGCAGATC	Servicebio	NM_001867.3
PRDX5-F:GATTCGCTGGTGTCCATCTTTG PRDX5-R:GTGCCATCTGGTTCCACATTCA	Servicebio	NM_001358511.2

**Software and algorithms**		

GraphPad Prism	GraphPad 10	https://www.graphpad.com
Cytoscape	Cytoscape 3.10.4	https://cytoscape.org
MSigDB	MSigDB2025.1 GSEA 4.4.0	https://www.gsea-msigdb.org/gsea/msigdb/index.jsp
R Studio	The R foundation	http://www.broadinstitute.org/mitocarta
MitoCarta	MitoCarta3.0	http://www.broadinstitute.org/mitocarta
STRING	STRING12.0	https://cn.string-db.org/
JASPAR	JASPAR2026	https://jaspar.elixir.no/

### Experimental model and study participant details

Species: Homo sapiens.

Sample Type: Serum.

Age: Adult participants.

Sex: 17 male, 13 female. Both male and female participants were included in this study. The sex distribution across groups is detailed in Table 1.

**Table 1:** The clinical characteristics of samples

Characteristics	COPD group (*n* = 9)	NSCLC group (*n* = 8)	NSCLC with COPD group (*n* = 6)	Control group (*n* = 7)
**Sex**				

Male	7 (77.8%)	4 (50%)	4 (66.7%)	2 (28.6%)
Female	2 (22.2%)	4 (50%)	2 (33.3%)	5 (71.4%)

**Age, years**				

Mean (SD)	59.0 (16.17)	60.38 (12.66)	65.0 (10.31)	29.71 (8.77)
Median (IQR)	61 (56.5–70.5)	59 (54.0–69.8)	68 (54.5–70)	34 (21–37)

**Smoker pack-years**				

Mean (SD)	30.0 (6.32)	30.0 (10.0)	43.33 (5.77)	5.0 (NaN)
Median (IQR)	30.0 (25.0–30.0)	30.0 (20.0–40.0)	40.0 (40.0–50.0)	5.0 (NaN)
Missing	3 (33.3%)	5 (62.5%)	3 (50.0%)	6 (85.7%)

**GOLD stage**				

Mean (SD)	3.0 (0.87)	0 (0%)	1.67 (0.52)	0 (0%)
Median (IQR)	3.0 (2.0–3.75)	0 (0%)	2.0 (1.0–2.0)	0 (0%)
Missing	0 (0%)	0 (0%)	0 (0%)	0 (0%)
TNM staging	–	–	–	–
Mean (SD)	–	1.75 (0.71)	5.0 (2.19)	–
Median (IQR)	–	2.0 (1.0–2.0)	5.0 (3.0–7.0)	–
Missing	–	0 (0%)	0 (0%)	–
Comorbidities (type II respiratory failure)	–	–	–	–
Mean (SD)	1.5 (0.54)	–	–	–
Median (IQR)	1.5 (1.0–2.0)	–	–	–
Missing	0 (0%)	–	–	–

Ancestry, Race, and Ethnicity Information: All participants self-reported Asian ancestry, specifically Chinese Han ethnicity. In this study, race and ethnicity information was collected and described based on participants’ self-identification.

Maintenance/Husbandry Conditions: Not applicable.

Institutional Permissions and Regulations: This study obtained approval from the Ethics Committee of the Second Hospital of Hebei Medical University, with an Ethics Approval Number of 2023-C062. All participants provided written informed consent prior to inclusion in the study.

#### Sample allocation

A total of 30 serum samples from eligible adult participants were included in this study. All samples were allocated according to the criteria described in Section “method details sample collection”. Specifically, COPD group (*n* = 9), NSCLC group (*n* = 8), NSCLC with COPD group (*n* = 6), Control group (*n* = 7).

This study was not specifically designed to assess the impact of sex on the outcomes. Although participants included both males and females, subgroup analysis based on sex was not conducted due to limitations in sample size, which constitutes a limitation of this research. Future studies should include more balanced samples to explore potential sex-based differences.

### Method details

#### Sample collection

We collected serum samples and clinical information from COPD patients, NSCLC patients, NSCLC with COPD patients, and healthy individuals. The blood samples were drawn from patients diagnosed with COPD, NSCLC and NSCLC with COPD in the Department of Pulmonary and Critical Care Medicine of the Second Hospital of Hebei Medical University from January 2024 to December 2024, including 9 COPD patients, 8 NSCLC patients, 6 NSCLC with COPD patients, and 7 healthy volunteers. This study obtained approval from the Ethics Committee of the Second Hospital of Hebei Medical University, with an Ethics Approval Number of 2023-C062. Healthy volunteers were selected from those who underwent medical examinations at the Second Hospital of Hebei Medical University from January 2024 to December 2024. Patients agreed to participate in the study and signed a written informed consent form. ① Healthy volunteers: normal lung function; no pulmonary, cardiac, cerebral, hepatic or renal disease; no malignancy; no respiratory illness within the past 2 months; and no history of smoking. ② COPD inclusion criteria: Diagnosis consistent with the GOLD 2023 guidelines^49^; chronic cough, sputum production and dyspnoea; relevant risk factors (e.g., smoking, occupational dust, biomass fuel exposure); post-bronchodilator FEV1/FVC <0.70 and negative bronchodilator reversibility test; and exclusion of other disorders that could explain cough, sputum or dyspnoea. ③ NSCLC without COPD inclusion criteria: Pathologically confirmed non-small cell lung cancer according to IASLC TNM staging (8th edition)^50^; post-bronchodilator FEV_1_/FVC >0.70 with negative reversibility test. ④ NSCLC with COPD inclusion criteria: simultaneous fulfilment of criteria ② and ③. Patients with uncontrolled hypertension (BP > 160/100 mmHg despite treatment) were excluded; Patients with diabetes mellitus were excluded; Patients with other significant comorbidities that could affect mitochondrial function (chronic kidney disease, heart failure, liver cirrhosis, active autoimmune diseases) were excluded. All participants provided written informed consent prior to enrollment.

After blood sample collection, red blood cell lysis buffer (at 3 times the volume of the samples) was added to the EDTA anticoagulated blood samples and the mixture was inverted to mix. The samples were allowed to stand at room temperature for 5 min, with gentle mixing a few times during this period. Centrifugation was performed at 10,000 rpm for 1 min, and the supernatant was removed, leaving the white blood cell pellet. An appropriate amount of TRIzol reagent was added to the isolated white blood cells, the mixture was mixed well, and then frozen in liquid nitrogen. The samples were stored at −80°C and transported on dry ice.

#### Transcriptome sequencing and data quality control

##### Sample preparation and quality control

RNA Extraction: Extract total RNA using Trizol, column-based method, or magnetic bead method. Quality Control Testing: Concentration determination: Quantification using a Qubit fluorometer. Integrity detection: Analysis with Agilent 2100 Bioanalyzer (an RNA Integrity Number [RIN] of ≥6 or the nucleic acid content of RNA is greater than or equal to 200 ng is preferred).

RNA Enrichment and Fragmentation: mRNA Enrichment: For eukaryotes, enrich polyA-tailed mRNA using oligo (dT) magnetic beads. For prokaryotes or samples with low mRNA abundance, use an rRNA removal kit. RNA Fragmentation: Fragment RNA to 200–500 bp using chemical methods (high temperature + magnesium ions) or enzymatic digestion.

##### Reverse transcription and double-stranded cDNA synthesis

First-Strand Synthesis: Synthesize cDNA using reverse transcriptase with fragmented RNA as the template and random primers or oligo (dT) primers. Second-Strand Synthesis: Synthesize the second strand using DNA polymerase, often incorporating dUTP to preserve strand specificity (optional).

Core Steps of Library Construction: End Repair and A-Tailing: Repair cDNA ends to form blunt ends and add an A base to the 3' end (for TA ligation). Adapter Ligation: Ligate Illumina adapters with a T base (containing Index sequences for multiplex sequencing). Notes: Adapters should be thawed in advance and avoid repeated freeze-thaw cycles. Adjust adapter concentration according to the initial RNA amount (e.g., 1.5 μM adapters for 100 ng RNA). Library Purification: Remove unligated adapters and retain target fragments using AMPure XP beads or equivalent products.

##### Library amplification and Final Purification

PCR Amplification: Amplify with high-fidelity enzymes for 10–15 cycles to enrich effectively ligated fragments. Final Purification: Sort target fragments (e.g., 300–400 bp) using beads to remove primer dimers or small fragments.

##### Library quality control and sequencing

Quality Control: Detect fragment distribution using Agilent 2100 and quantify with Qubit. Sequencing on Machine: Perform PE150 sequencing on the Illumina NovaSeq platform. The library construction kit is VAHTS Universal V8 RNA-seq Library Prep Kit for Illumina.

After obtaining the raw sequencing data (Pass Filter Data), the following process is carried out for bioinformatics analysis: Obtain the raw data, perform quality control on the raw data, conduct reference genome alignment through SNV analysis and DEU analysis, then proceed with advanced analyses (including gene fusion analysis, RNA editing, co-expression network analysis, key gene screening, LncRNA prediction and functional analysis, protein interaction network, GSEA, time-series analysis, transcription factor annotation, etc.). At the same time, perform transcript assembly based on the reference genome alignment results, followed by differential alternative splicing analysis, and continue with new transcript prediction and gene expression analysis. Subsequently, perform an overall RNA quality assessment (such as saturation analysis and uniformity analysis) and differential gene analysis (cluster analysis, GO enrichment analysis, and pathway enrichment analysis).

#### Identification of differentially expressed genes (DEGs) and gene sets

Differential expression analysis was performed using the DESeq2 package in R, comparing control samples against COPD, NSCLC, and NSCLC with the COPD groups separately. Significant DEGs were identified using adjusted *p*-value <0.05 and absolute log2 fold change >1 as thresholds. The DEGs obtained from the comparisons between control and COPD, control and NSCLC, and control and NSCLC with COPD were defined as DEG1, DEG2, and DEG3, respectively. For the identification of differentially expressed gene sets, we downloaded the Hallmark gene sets from the Molecular Signatures Database (MSigDB) as reference. Gene Set Variation Analysis (GSVA) was implemented to calculate enrichment scores for each gene set across samples.^51^ The R package “limma” was employed to identify differentially expressed gene sets between control samples and COPD, NSCLC, or NSCLC with COPD samples with significance thresholds of *p* < 0.05 and absolute t-statistic >2.^52^

#### Functional enrichment analysis

Gene Ontology (GO) analysis and Kyoto Encyclopedia of Genes and Genomes (KEGG) pathway analysis, were conducted using clusterProfiler.^53^ For both GO enrichment and KEGG pathway enrichment, significantly enriched terms were identified with an adjusted *p*-value <0.05.

#### Screening of MRGs and common genes

A curated set of 1,136 MRGs was obtained from MitoCarta 3.0(http://www.broadinstitute.org/mitocarta).^54^ We obtained mitochondria-related DEGs in COPD, NSCLC, and NSCLC with COPD by intersecting DEG1, DEG2, and DEG3 with MRGs, respectively. Common gene sets were derived by intersecting COPD-MRGs with NSCLC with COPD-MRGs (Common Gene Set 1) and NSCLC-MRGs with NSCLC with COPD-MRGs (Common Gene Set 2).

#### Construction of protein-protein interaction networks for common genes

Common Gene Set 1 and Common Gene Set 2 were separately input into the STRING database with an interaction score threshold of 0.4 to construct protein-protein interaction (PPI) networks. These networks were visualized using Cytoscape,^55^ and the top 10 genes were selected via the MCC algorithm as Candidate Common Gene Set 1 and Candidate Common Gene Set 2, respectively.

#### Machine learning-based biomarker identification

Support Vector Machine-Recursive Feature Elimination (SVM-RFE) was applied to recognize diagnostic biomarkers from Candidate Common Gene Set 1 and Candidate Common Gene Set 2. The R package mlbench was used for automated screening of candidate Common Gene Set 1 to identify biomarkers capable of distinguishing control from COPD samples and control from NSCLC with COPD samples. The intersection of these two sets of biomarkers was defined as Biomarker Set 1, Similarly, Candidate Common Gene Set 2 was screened to identify biomarkers for distinguishing NSCLC vs. control and NSCLC with COPD vs. control, and their intersection was defined as Biomarker Set 2.

#### Diagnostic performance evaluation of biomarkers sets

For Biomarker 1, transcriptome datasets (COPD vs. control and NSCLC with COPD vs. control) underwent Receiver Operating Characteristic (ROC) analysis, with the analysis implemented using the R package pROC.^56^ For Biomarker 2, ROC curve analysis was conducted on the datasets (NSCLC vs. control and NSCLC with COPD vs. control). Using multivariate logistic regression via the R package rms, a scoring system was created relying on the expression levels of Biomarker Set 1 and Biomarker Set 2, where each factor was assigned a score, and the total score (TotalPoint) corresponded to the sum of these individual scores. This total score was then applied to forecast the probability of disease diagnosis, with higher scores indicating a higher probability of being diagnosed with the disease. Nomograms, calibration curves, and decision curve analyses (DCA) were generated for clinical utility to evaluate the association between Biomarker 1 and COPD/NSCLC with COPD risk, as well as between Biomarker 2 and NSCLC/NSCLC with COPD risk.

#### Single-gene GSEA analysis of biomarkers

Spearman correlation analysis was performed between each gene in Biomarker Set 1 and Biomarker Set 2 and to obtain correlation coefficients, all other genes were processed using the R package corrplot. After sorting by correlation coefficients, Gene Set Enrichment Analysis (GSEA) was performed with the R package clusterProfiler, utilizing the c2.cp.kegg.v2023.2.Hs.symbols.gmt gene set from MSigDB that comprises 186 gene sets.^57^

#### Immune infiltration analysis

The R package CIBERSORT served to score the abundances of 22 immune cell types within the training set.^58^ Wilcoxon tests were performed to identify immune cells with notably distinct infiltration abundances when comparing COPD vs. control, NSCLC vs. control, and NSCLC with COPD vs. control, with a significance threshold of *p* < 0.05. Spearman correlation analyses were conducted to assess correlations between immune cells, between immune cells and Biomarker Set 1, and between immune cells and Biomarker Set 2, with the criteria of absolute correlation coefficient (|Cor|) > 0.3 coupled with *p* < 0.05.

#### Interaction of biomarkers with diseases, establishing regulatory networks and identifying potential drugs

The Comparative Toxicogenomics Database (CTD) database was used to evaluate the associations between Biomarker Set1 and COPD/NSCLC with COPD, as well as between Biomarker Set2 and NSCLC/NSCLC with COPD.^59^ Transcription factors (TFs) targeting Biomarker Set1 and Biomarker Set2 were predicted from the Jaspar database via the NetworkAnalyst platform.^60^ Drugs targeting Biomarker Set1 and Biomarker Set 2 were predicted using the DGIdb database,^61^ and all networks were visualized in Cytoscape.

#### Western blot analysis

We collected the whole blood sample of 7 control patients, 9 COPD patients, 8 NSCLC patients, and 6 NSCLC with COPD patients for Western blot analysis. First, whole human blood is lysed in red blood cell lysis buffer, and then centrifuged (3000 rpm, 5 min) to remove the red blood cells. The supernatant is discarded, leaving the white blood cell pellet, to which RIPA lysis buffer is added for lysis. After cells are lysed by RIPA buffer, total proteins in cell lysates are extracted. Then, the protein concentration is detected by NanoDrop 2000C spectrophotometer. Next, the total proteins are loaded into 12% SDS-PAGE gels. After electrophoretic separation of proteins, they are transferred to a 0.45 μm PVDF membrane. Subsequently, after blocking in 5% BSA for 1 h, the membrane is incubated overnight at 4°C with NDUFB6 (1:5000, CUSABIO, China), UQCRB (1:2000, Abiowell, China), PRDX5 (1:2000, Abiowell, China), BID (1:2000, Abiowell China). After washing three times with TBST, the membrane is incubated with the secondary antibody at room temperature for 1 h. After washing, screening and visualization are performed using an ECL chemiluminescence imager. Protein levels are normalized to GADPH (1:10000,Abways, China).

### Quantification and statistical analysis

Statistical analyses were performed using R software (version 4.4.1). For comparisons between two groups, the Wilcoxon rank-sum test (Mann-Whitney U test) was used. For comparisons among multiple groups, the Kruskal-Wallis’s test followed by Dunn’s post-hoc test was applied. Correlation analyses were performed using Spearman’s correlation coefficient. For diagnostic model evaluation, receiver operating characteristic (ROC) curves were generated and the area under the curve (AUC) with 95% confidence intervals was calculated. Model calibration was assessed using 2000 bootstrap resamples. Decision curve analysis (DCA) was performed to evaluate the clinical utility of the prediction models. Statistical significance was defined as *p* < 0.05. In all figures, statistical significance is denoted as follows: ∗*p* < 0.05, ∗∗*p* < 0.01, ∗∗∗*p* < 0.001, ∗∗∗∗*p* < 0.0001; ns, not significant. All data are presented as median with interquartile range unless otherwise specified.
