# Supplementary material for: Machine learning-driven identification of shared and disease-specific mitochondria-related genes in COPD, NSCLC, and NSCLC with COPD
Source: iScience. 2026 Jan 29;29(3):114857. doi: 10.1016/j.isci.2026.114857 (PMC12925241; doi:10.1016/j.isci.2026.114857)
Supplement: Document S1. Figures S1–S5 [file mmc1.pdf]

iScience, Volume *29*

Supplemental information

Machine learning-driven identification of shared and disease-specific mitochondria-related genes in COPD, NSCLC, and NSCLC with COPD

Siyu Wu, Zelin Chen, Tongxinwei Sun, Beibei Song, Xinxiu Liu, Liwen Zhang, Jing Li, Haoran Lu, Wenhui Song, and Aihong Meng

Supplementary Figure 1

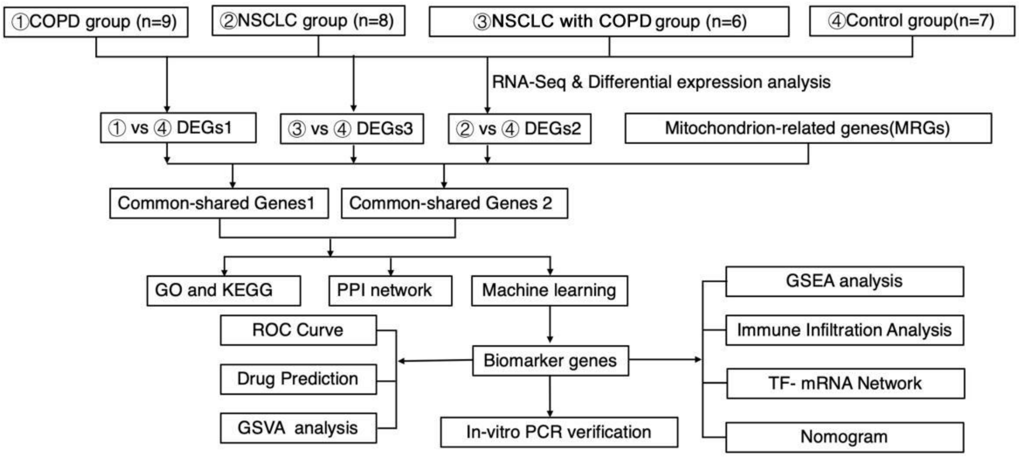

Supplementary Figure 1. The flowchart of this study.

Supplementary Figure 2

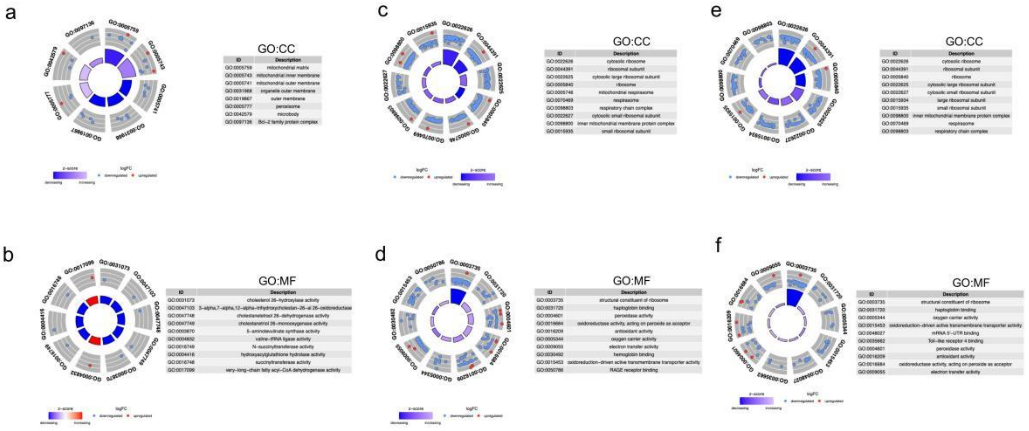

Supplementary Figure 2. GO analysis of DEGs. The top ten cell component terms of GO analysis of the DEGs1 between COPD and control group (a), DEGs2 between NSCLC and control group(c), DEGs3 between NSCLC with COPD and control group(e). The top ten molecular function terms of GO analysis of DEGs1 between COPD and control group (b), DEGs2 between NSCLC and control group(d), DEGs3 between NSCLC with COPD and control group(f).

Supplementary Figure 3

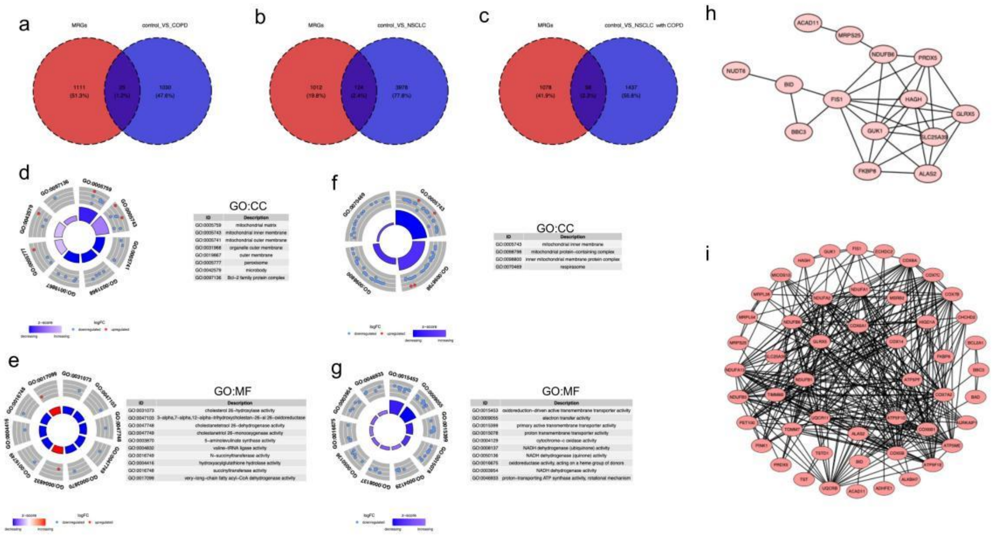

Supplementary Figure 3. Identification and functional characterization of common genes. (a-c) Intersection of MRGs with (a) DEG1 (COPD-associated), (b) DEG2 (NSCLC-associated), and (c) DEG3 (NSCLC with COPD - associated) , respectively. (d&f) Cellular component (CC) terms from GO analysis of Common Genes Set 1 (d) and Common Genes Set 2 (f). (e&g) Molecular function (MF) terms from GO analysis of Common Genes Set 1 (e) and Common Genes Set 2 (g). (h–i) Protein–protein interaction (PPI) networks of Common Genes Set 1 (h) and Common Genes Set 2 (i).

Supplementary Figure 4

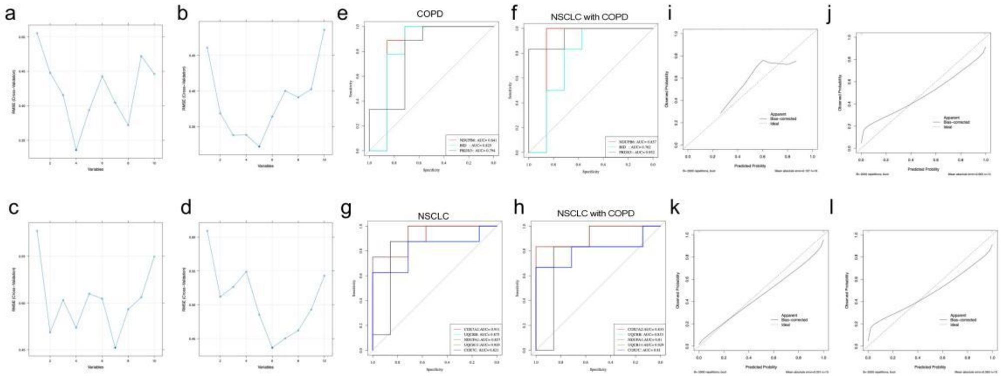

Supplementary Figure 4. Biomarker screening and diagnostic model evaluation.
(a-b) SVM-RFE feature selection for biomarkers distinguishing COPD vs controls (a) and NSCLC with COPD vs controls (b) from Common Genes Set 1. (c-d) SVM-RFE selection for biomarkers differentiating NSCLC vs controls (c) and NSCLC with COPD vs controls (d) from Common Genes Set 2. (e-f) ROC curve evaluation of diagnostic performance for control vs COPD (e) and control vs NSCLC with COPD (f). (g-h) ROC curve evaluation of diagnostic performance for control vs NSCLC (g) and control vs NSCLC with COPD (h). AUC values with 95% confidence intervals are shown in the legends. (i-j) Calibration curves for Biomarker 1 in COPD (i) and NSCLC with COPD (j) prediction. (k-l) Calibration curves for Biomarker 2 in NSCLC (k) and NSCLC with COPD (l) prediction.

Supplementary Figure 5

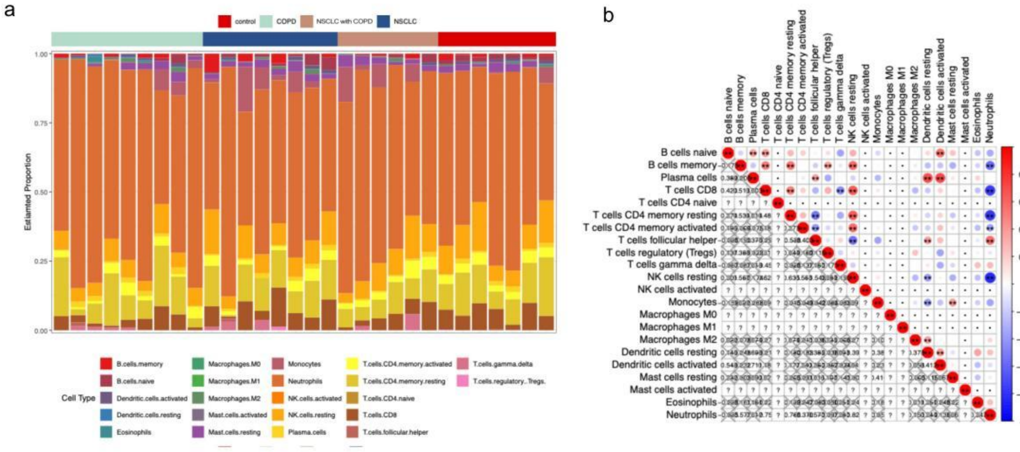

Supplementary Figure 5. Immune cell composition and correlation patterns. (a) Stacked bar plot showing relative abundances of 22 immune cell types across samples. (b) Correlation heatmap of immune cell type infiltration levels.

Data S1: Original Western Blot Data from This Study

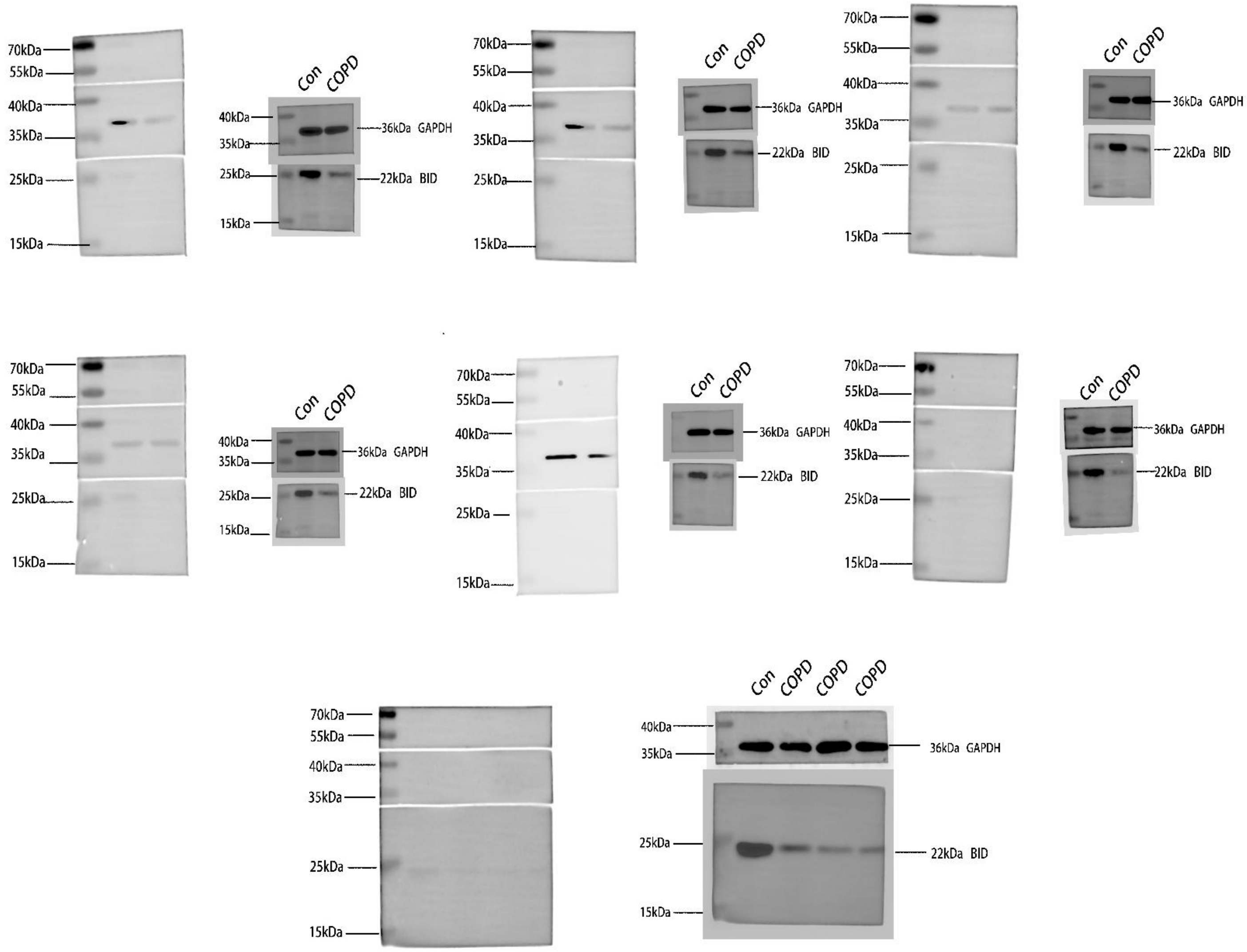

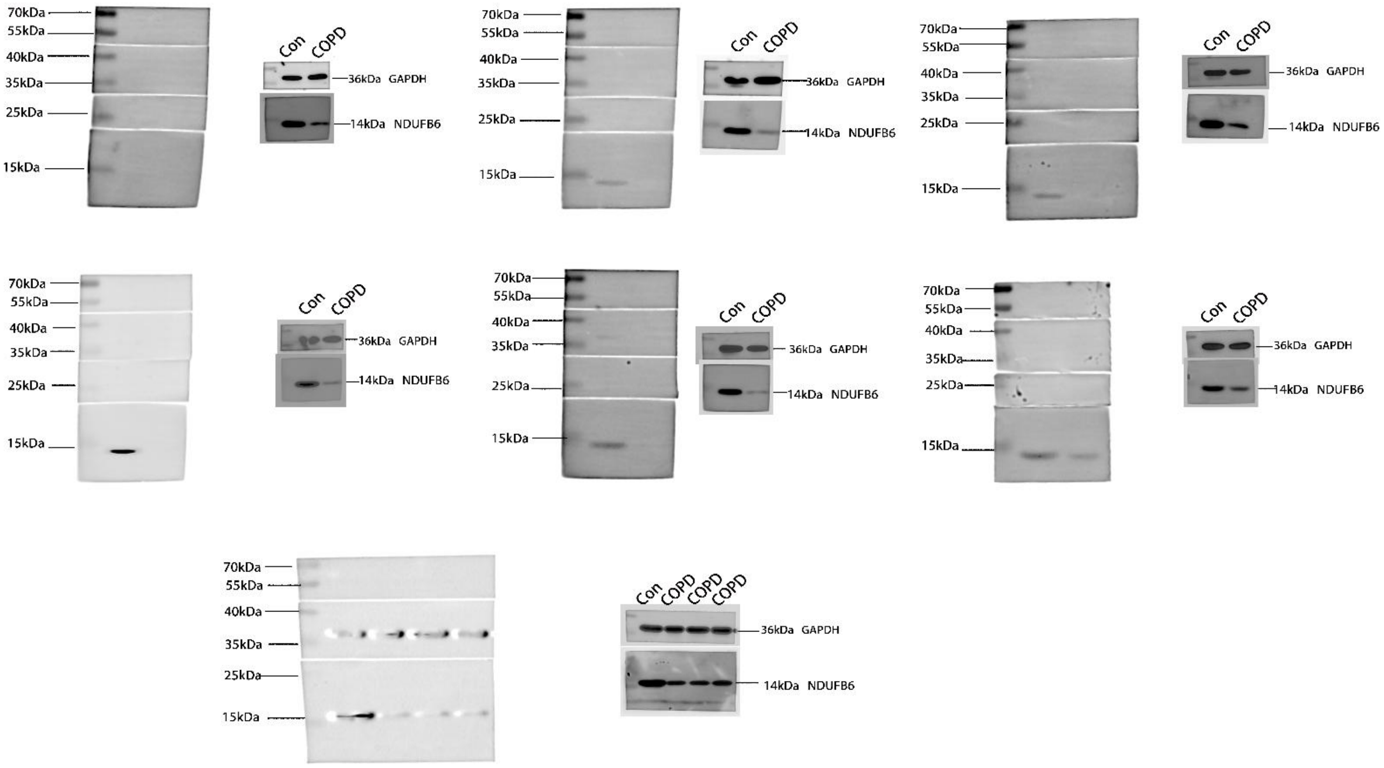
70kDa
55kDa
40kDa
35kDa
25kDa
15kDa
Con
COPD
36kDa GAPDH
14kDa NDUFB6

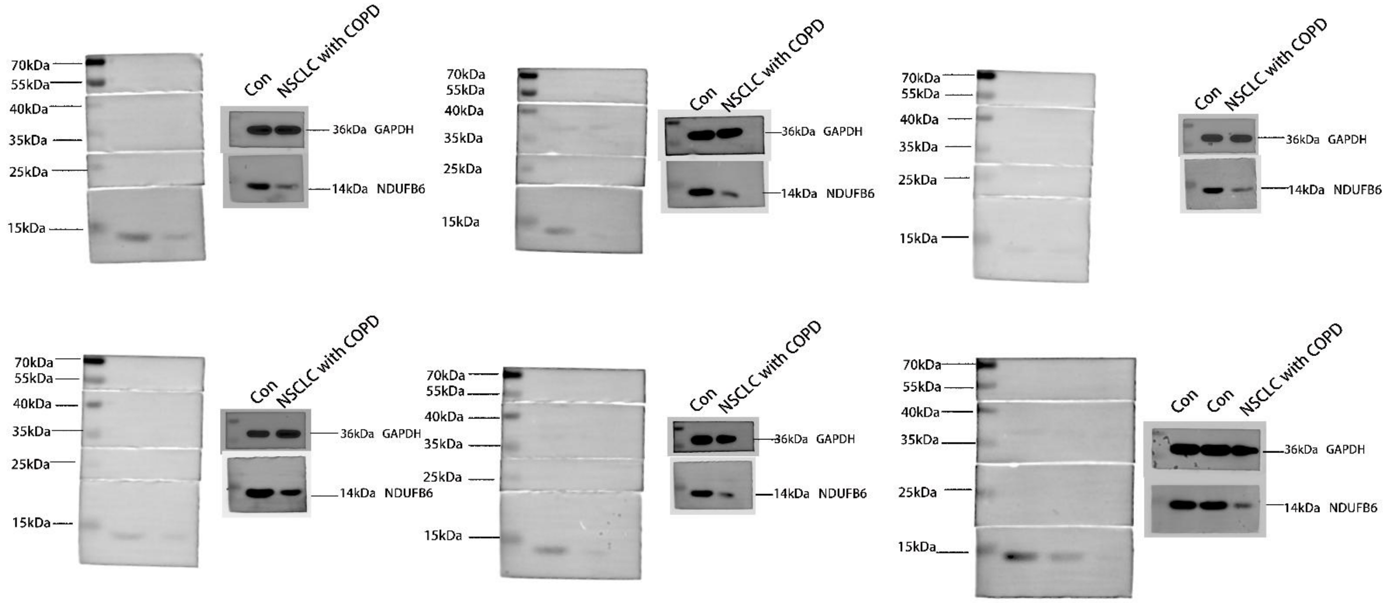
70kDa
55kDa
40kDa
35kDa
25kDa
15kDa
Con
NSCLC with COPD
36kDa GAPDH
14kDa NDUFB6

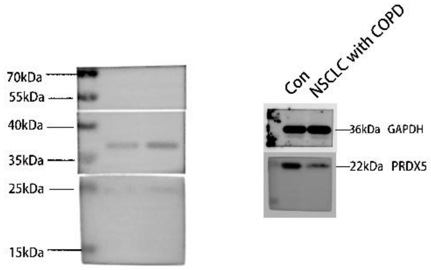
70kDa
55kDa
40kDa
35kDa
25kDa
15kDa
Con
NSCLC with COPD
36kDa GAPDH
22kDa PRDX5

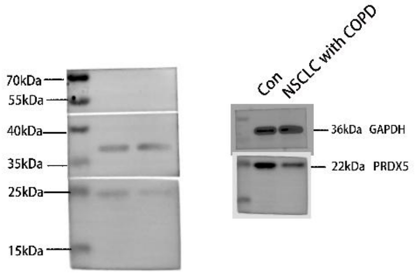
70kDa
55kDa
40kDa
35kDa
25kDa
15kDa
Con
NSCLC with COPD
36kDa GAPDH
22kDa PRDX5

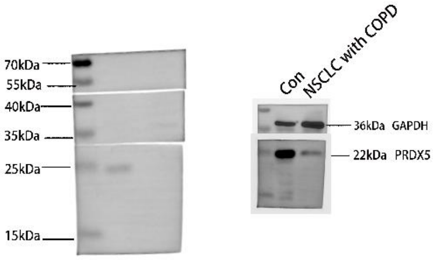
70kDa
55kDa
40kDa
35kDa
25kDa
15kDa
Con
NSCLC with COPD
36kDa GAPDH
22kDa PRDX5

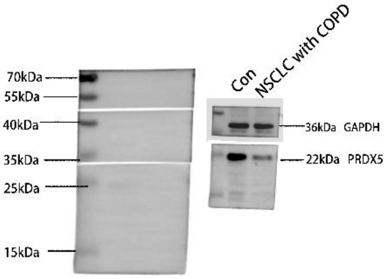
70kDa
55kDa
40kDa
35kDa
25kDa
15kDa
Con
NSCLC with COPD
36kDa GAPDH
22kDa PRDX5

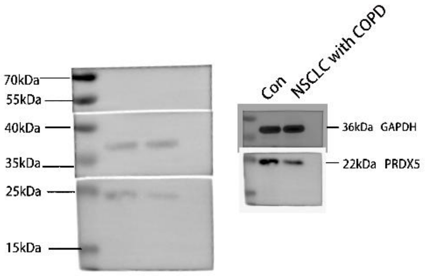
70kDa
55kDa
40kDa
35kDa
25kDa
15kDa
Con
NSCLC with COPD
36kDa GAPDH
22kDa PRDX5

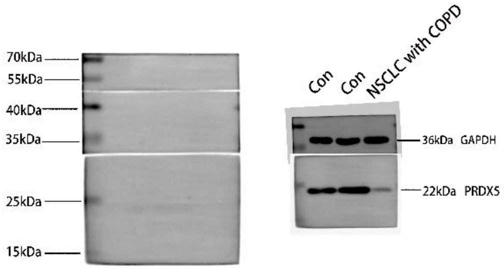
70kDa
55kDa
40kDa
35kDa
25kDa
15kDa
Con
Con
NSCLC with COPD
36kDa GAPDH
22kDa PRDX5

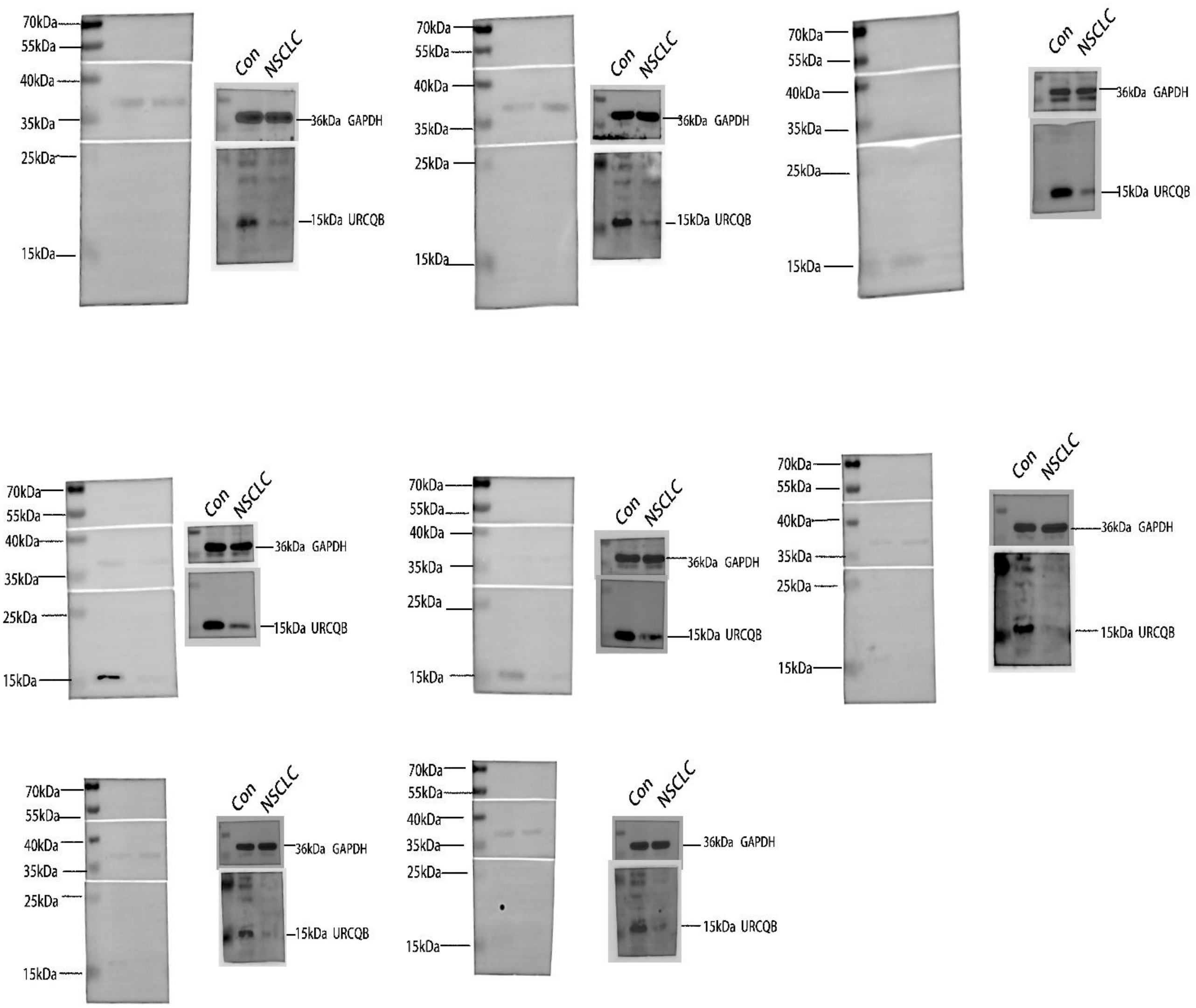

70kDa
55kDa
40kDa
35kDa
25kDa
15kDa
Con
NSCLC
36kDa GAPDH
15kDa URCQB
70kDa
55kDa
40kDa
35kDa
25kDa
15kDa
Con
NSCLC
36kDa GAPDH
15kDa URCQB
70kDa
55kDa
40kDa
35kDa
25kDa
15kDa
Con
NSCLC
36kDa GAPDH
15kDa URCQB
70kDa
55kDa
40kDa
35kDa
25kDa
15kDa
Con
NSCLC
36kDa GAPDH
15kDa URCQB
70kDa
55kDa
40kDa
35kDa
25kDa
15kDa
Con
NSCLC
36kDa GAPDH
15kDa URCQB
70kDa
55kDa
40kDa
35kDa
25kDa
15kDa
Con
NSCLC
36kDa GAPDH
15kDa URCQB
70kDa
55kDa
40kDa
35kDa
25kDa
15kDa
Con
NSCLC
36kDa GAPDH
15kDa URCQB
70kDa
55kDa
40kDa
35kDa
25kDa
15kDa
Con
NSCLC
36kDa GAPDH
15kDa URCQB
